# Revision of the Chinese species of subgenus Koreonialoe (Coleoptera: Carabidae: *Pterostichus*), with descriptions of two new species

**DOI:** 10.3897/zookeys.1063.69942

**Published:** 2021-10-18

**Authors:** Wen-Qi Yin, Hong-Liang Shi, Hong-Bin Liang

**Affiliations:** 1 College of Forestry, Beijing Forestry University, Beijing 100083, China Beijing Forestry University Beijing China; 2 Key Laboratory of Zoological Systematics and Evolution, Institute of Zoology, Chinese Academy of Sciences, Beijing 100101, China Institute of Zoology, Chinese Academy of Sciences Beijing China

**Keywords:** Endophallus, genitalia, ground beetles, new species, Northeast China

## Abstract

The Chinese species of subgenus Koreonialoe Park & Kwon, 1996 of the genus *Pterostichus* are revised, including four species from the eastern part of Jilin and Liaoning provinces. Two new species are described: Pterostichus (Koreonialoe) micropoides**sp. nov.** (type locality: Jilin, Changbai county), and Pterostichus (Koreonialoe) tetralobatus**sp. nov.** (type locality: Liaoning, Xiuyan county). Pterostichus (Koreonialoe) bellatrix (Tschitschérine) is newly recorded from China (Jilin). The subgenus Koreonialoe is classified into two groups on account of their differences on the endophallus, and all Chinese species accord with the *microps* group defined herein. A key to all six species in the *microps* group is provided.

## Introduction

The subgenus Koreonialoe includes a group of pterostichine beetles with large heads, elongate mandibles, opaque elytra, and the male sternum unmodified. They are distributed in the Korean Peninsula and adjacent areas (including the Northeast China, Primorskiy Kray, and Tsushima). This group was initially recognized as the *opacipennis* group under subgenus Nialoe by Nemoto (1988), who revised this group containing seven species from the Korean Peninsula and Tsushima (Japan). Park and Kwon (1996) erected the subgenus Koreonialoe to accommodate species in the *opacipennis* group and described three species and one subspecies from South Korea. The first Chinese species in this group, *P.syleus* was described by Kirschenhofer (1997), but this species was initially placed in the Nialoe until Sasakawa et al. (2013) assigned it to the subgenus Koreonialoe. Subsequently, three new species were described (Sasakawa et al. 2008; Li and Zhang 2014), and *P.togyusanus* was upgraded to the species level (Park et al. 2013) from a subspecies of *P.bellatrix*.

The subgenus Natalianoe was erected by Berlov and Plutenko (1997) for its type species *P.microps* Heyden (distributed in Russia, Primorskiy Kray) and other similar macrocephalic species from Korea and Japan. Most of these species mentioned by Berlov and Plutenko (1997) are now placed in subgenus Koreonialoe and in the *P.macrogenys* group of *Nialoe* (Sasakawa 2005b). *Pterostichusmicrops* is morphologically very similar to some species in *Koreonialoe* and was placed in *Koreonialoe* by Lafer (2005) and Sundukov (2013), but it was not treated in recent important revisions (Sasakawa et al. 2013; Park and Park 2013).

Prior to the present study, there was a total of 16 species in *Koreonialoe*, with one species from Russia, Primorskiy Kray (*P.microps*), one from Japan, Tsushima (*P.opacipennis*), one from China, Liaoning (*P.syleus*), and the other 13 from the Korean Peninsula. The single Chinese *Koreonialoe* species, *P.syleus* has been rarely collected and is known only from its type locality, Sifangding, Liaoning. During expeditions to Northeast China in recent years, a large number of specimens belonging to this subgenus were collected from different localities of the eastern part of Jilin and Liaoning provinces (Fig. 36). Although these specimens are almost identical in their external features, detailed studies on the endophallus of male genitalia show that four different species are present in China: two of them were undescribed and *P.bellatrix* is recorded from China for the first time.

The present study describes two new species from Northeast China, newly records *P.bellatrix* from China (Jilin), and provides a key for all six species with ventrally directed endophallus of male genitalia. All new species are amply described and illustrated, especially for their endophallic characters.

## Materials and methods

The present study is mainly based on the examination of specimens from Northeast China. All specimens examined, including types of new species, are deposited in the collections of the Institute of Zoology, Chinese Academy of Sciences, Beijing, China (IZAS).

The body length (BL) was measured from the apical margin of the labrum to the elytral apex; body width (BW) was measured along the elytral greatest width; the width of head (HW) was measured along the greatest transverse distance of head including eyes. The pronotum width (PW) was measured along its greatest width; pronotum length (PL) was measured along its median line; basal width (PBW) was measured along its posterior margin; apical width (PAW) was measured along its apical margin. Elytra length (EL) was measured along the suture from the base of the scutellum to the elytra apex.

The terminology of the endophallic lobes of male genitalia follows Sasakawa et al. (2005a, 2013). The abbreviations used in the endophallus are as follows: left apical lobe (**la**, = lobe β in Nemoto 1988; Park and Kwon 1996), left preapical lobe (**lp**, = lobe α (part) in Nemoto 1988; Park and Kwon 1996), right apical lobe (**ra**), gonopore (**gp**), gonopore lobe (**gpl**). In some species from China, the median lobe of aedeagus has a tubercle (**tu**) on the left ventral side. Other terms used, dissection techniques, endophallus everting procedures, and photography follow Shi et al. (2013).

## Taxonomy

### Subgenus Koreonialoe

Taxon classificationAnimaliaColeopteraCarabidae

Park & Kwon, 1996

EA14ED7F-8110-5EC7-A41F-32F98E45D290

Koreonialoe Park & Kwon, 1996: 93. Type species: Pterostichusteretis Park & Kwon, by original designation. Park and Park 2013: 66. Bousquet 2017: 717.
Natalianoe Berlov & Plutenko, 1997: 4. Type species: Pterostichusmicrops Heyden, by original designation. Sundukov 2013: 130 (as junior synonym of Koreonialoe).
“opacipennis species-group” of Nialoe: Nemoto 1988: 39. Sasakawa et al. 2008: 174. Sasakawa et al. 2013: 430.

#### Diagnosis.

Medium to large-sized *Pterostichus*; head large and thick, with elongate mandibles; eyes small, temporae swollen, a little wider than width across outer margins of eyes; pronotum cordate with posterior angles nearly rectangular; elytra entirely opaque; sternite VI or VII of males without secondary sexual modification.

#### Comparison.

*Koreonialoe* species all share two external morphological features not found in other *Nialoe* species (= *Nialoesensu lato*: Kasahara 1988; Sasakawa 2005a): elytra entirely opaque from dense, isodiametric microsculpture; males without secondary sexual modification of sternite VII. *Koreonialoe* is considered to be a clade sister to the *macrogenys* species group (Sasakawa 2005b). These two groups are similar in their enlarged head with elongate mandibles and swollen temporae. But the latter group differs in the dorsal surface of elytra shiny and sternite VII of males is more or less concave.

#### Subgeneric characters.

Body robust, medium to large size, body length 12–25 mm, elongate and macrocephalic. Dorsally black, dark brown or reddish brown; pronotum finely punctate, more or less opaque; elytra entirely opaque, without metallic luster. Palpi and tarsi brown, other parts of legs similar color as elytra. Head large and thick; eyes small and convex, tempora strongly swollen, generally a little longer than length of eyes; frons with shallow and sparse punctures throughout; frontal grooves shallow and wide, reaching midpoint of eyes. First antennomere shorter than combined length of following two segments. Mandibles long, outer surface nearly straight, apex hooked; apical margin of labrum and clypeus deeply emarginate; terminal segment of labial palpus fusiform, a little dilated in males; mentum tooth bifid, mentum with a pair of longitudinal depressions, submentum with two setae on each side. Pronotum strongly cordate, widest near anterior third, one-sixth to one-fifth wider than length. Surface more or less opaque, with fine punctures, sub-anterior transversal sulci well defined, strongly curved. Lateral margins slightly arched from anterior angles to the middle, strongly sinuate and then nearly straight before posterior angles; posterior angles nearly rectangular; posterior margin strongly emarginate at middle. Mid-lateral setae present near anterior fifth of lateral margins; lateral expansions narrow, equally wide at anterior and posterior portions. Basal foveae with inner and outer grooves faintly defined and partly fused, forming deep depression between them; outer groove slightly shorter than inner one (definition of pronotal inner and outer grooved see Shi et al. 2013: 103); middle area between two basal foveae with strong transverse wrinkles or heavy punctures. Elytra entirely opaque, length a little greater than one-half of width, widest a little behind middle. Shoulders widely rounded; basal ridge and lateral margins forming obtuse angle; striae regular, parascutellar dorsal pore present on the end of first stria; two or three discal pores on third interval, all adjoining to second stria. Umbilicate series on ninth interval continuous, sparse at middle. Ventral side: metepisternum slightly shorter than basal width, nearly smooth; stermite VII with one seta on each side in males, with two in female, male sternite VI or VII without secondary sexual modification. Mesofemora with two setae; metacoxae with two setae; metatrochanters without setae; fifth tarsomere glabrous or setose ventrally. Male genitalia with median lobe of aedeagus stout, curved at basal one-third to one-fourth; right paramere short and straight. Endophallus straight or directed ventrally, with two or more lobes at anterior part. Female genitalia: Gonocoxite II of ovipositor falciform in ventral view, length ca. three times basal width; outer margin with two or three short and fine ensiform setae irregularly arranged, inner margin without ensiform setae; apex strongly compressed, rounded in lateral view, with two nematiform setae in a groove (Figs 28–31). Spermatheca with seminal canal and receptaculum differentiated, receptaculum digitate, shortly branched at base, seminal canal much slenderer than receptaculum, three to five times length of receptaculum; spermathecal gland inserted on the base of receptaculum (Figs 32–35).

#### Distribution.

A total of 18 species distributed along the Korean Peninsula and adjacent areas, including the eastern part of Jilin and Liaoning (China), Primorskiy Kray (Russia), Tsushima Island (Japan).

#### Remarks.

Different species of the subgenus Koreonialoe are extremely similar in their external appearances. All known species are almost indistinguishable from each other by external characters, and even difficult to differentiate by the sclerotized part of male genitalia. Thus previous species delimitation under this subgenus was mainly based on the male endophallic characters, especially the number, location, and shape of apical lobes (Nemoto 1988; Park and Kwon 1996; Sasakawa et al. 2008).

The endophallus of male genitalia is also important for inferring phylogeny (Sasakawa 2005a). Previous studies showed that most species (11 of 14) in *Koreonialoe* have their endophallus short and straight, but the remaining three species (*P.bellatrix*, *P.syleus*, and *P.togyusanus*) have the endophallus more elongate and clearly directed ventrally (Sasakawa et al. 2013). In the present study, we found that the ventrally directed and elongate endophallus present in all four species distributed in China, as well as in *P.microps* which was not treated by Sasakawa et al. (2013).

To better interpret the infra-subgeneric taxonomy of *Koreonialoe*, names of two groups are introduced: 1) the *opacipennis* group (*sensu stricto*, *nec.*Nemoto 1988): containing twelve species distributed in the Korean Peninsula and Tsushima, with a short and straight endophallus, gonopore that opens apically to the aedeagus, the ostium very weakly turn left. 2) the *microps* group: containing six species in Northeast China, the Korean Peninsula, and Primorskiy Kray, with elongate and ventrally directed endophallus, gonopore that opens basally to the aedeagus, aedeagal apex more or less deflected ventrally, the ostium more evidently turned left. Although these two groups included in the subgenus Koreonialoe are not based on a phylogenetic analysis, it seems likely that the *opacipennis* group (sensu strict) could be monophyletic for their highly specialized endophallus.

The determination of female specimens of *Koreonialoe* is very difficult sometimes. In most subgenera of *Pterostichus*, sibling species usually can be differentiated by the outline of pronotum, pronotal basal foveal characters, including the punctuation, length and depth of basal foveal grooves, chaetotaxy on elytra and legs, elytral striae depth and punctation, microsculpture, and male modification on the sternum. But in *Koreonialoe*, these important characters are always identical for most species. Moreover, the female ovipositor and reproductive tracts also do not help in species determination (Figs 28–35). In a few cases, the body size and color are helpful, for example in the two sympatric species *P.micropoides* and *P.bellatrix*, the latter one is always much larger and darker in color. But many allopatric species are usually extremely similar to each other. In these cases, females can be determined only through the males collected in exactly the same locality. Therefore, in the present study, the key to species is mainly based on the characters of male genitalia, and this key does not help determine females. But under each Chinese species, we provide comparisons to similar species on external features as well.

### Key to species of subgenus Koreonialoe (Part of males)

**Table:** 

1	Endophallus short and straight, gonopore opened apically on aedeagus (Sasakawa 2005: Fig. 3J); aedeagal apex usually not deflected ventrally, ostium very weakly turned left	***opacipennis* group**
–	Endophallus elongate and directed ventrally, gonopore opened basally to aedeagus (Figs 22–27); aedeagal apex more or less deflected ventrally, ostium more evidently turned left (***microps* group**)	**2**
2	Median lobe of aedeagus with ventral margin more or less tumid (Figs 9–12)	**3**
–	Median lobe of aedeagus with ventral margin not tumid (Figs 7, 8)	**5**
3	Median lobe of aedeagus with a large conic tubercle near middle of ventral margin (Fig. 12); apical portion of median lobe gradually deflected ventrally; left apical lobe of endophallus hooked	***P.bellatrix* Tschitschérine**
–	Median lobe of aedeagus slightly tumid before middle of ventral margin (Figs 9–11); apical portion abruptly deflected ventrally; left apical lobe of endophallus not hooked	**4**
4	Endophallus with four lobes, left apical lobe divided into two sub-lobes (Fig. 26)	***P.tetralobatus* sp. nov.**
–	Endophallus with three lobes, left apical lobe not divided (Figs 24, 25)	***P.syleus* Kirschenhofer**
5	Dorsally nearly black; endophallus with three lobes, right apical lobe present; left preapical lobe with apex conic (Sasakawa et al. 2013: Fig. 2A)	***P.togyusanus* Park & Kown**
–	Dorsally dark reddish brown; endophallus with two lobes, right apical lobe absent; left preapical lobe with apex spherical (Figs 21, 22, 23)	**6**
6	Endophallus directed ventrally; left apical lobe a little smaller than left preapical lobe; lp and la both spherical (Figs 22, 23)....	***P.micropoides* sp. nov.**
–	Endophallus directed dorsal-ventrally; left apical lobe strongly compressed, much smaller than left preapical lobe (Fig. 21)	***P.microps* Heyden**

### 1. Pterostichus (Koreonialoe) micropoides sp. nov.

Taxon classificationAnimaliaColeopteraCarabidae

9935E34F-28EE-5620-BD6E-C2521E242F05

http://zoobank.org/F3B14AEA-5732-494E-9CA9-BA042FEE15B9

Figs 1 , 2 , 7 , 8 , 13 , 14 , 22 , 23 , 30 , 34

#### Type locality.

China, Jilin province: Baishan city, Changbai county, Changsongling (41.74N, 128.02E, alt 1330 m).

#### Type material.

***Holotype*:** male, “Jilin province, Baishan City, Changbai County, E of Changsongling tunnel; mixed forest, 41.7398N, 128.0221E, 1330m”; “pitfall trap, 2019.VIII.9, Shi HL & Liu YZ lgt. Exp. BJFU 2019”; “HOLOTYPE ♂ Pterostichus (Koreonialoe) micropoides sp. nov., des. Yin & Shi. 2021” [red label]. ***Paratypes*** (a total of 164 males and 104 females): 1 male and 2 females, the same data as holotype but labeled as paratype; 2 males and 2 females, “Jilin province, Baishan City, Changbai County, Manjiang Changsong Village; mixed forest, 42.8527N, 127.8287E, 107m”; “pitfall trap; 2019.VIII.9, Liu YZ, Wang C, Zhu PZ & Wu JY lgt.”; 5 males and 5 females, “Jilin province, Yanbian City, Antu County, Laoling along G334, Anhe view point; 42.5153N, 128.6744E, 1300m”; “pitfall trap; 2019.VII.31-2019.VIII.2, Liu YZ, Wang C, Wu JY & Zhu PZ lgt.”; 1 male, “Jilin province, Hunchun City, Jingxin county, Daxiutiandong Village; fagus forest, 42.6448N, 130.3486E, 30m”; “pitfall trap; 2019.VIII.10, Shi HL & Liu YZ lgt.”; 1 male, “Jilin province, Antu County, north slope of Changbai mountain, Tianchi waterfall meadow; 42.0373N, 128.0544E, 1959m”; “2019.VIII.7, Shi HL, Liu YZ & Wang C lgt.”; 2 males and 1 female, “Jilin province, Antu county, north slope of Changbai mountain, W to the waterfall; alpine meadow, 42.0477N, 128.0517E, 2117m”; “pitfall trap; 2019.VIII.7, Shi HL, Liu YZ & Wang C lgt.”; 4 males and 1 female, “Jilin province, Baishan city, Fusong county, W of Changsongling tunnel; mixed forest, 41.7798N, 127.9400E, 1577m”; “pitfall trap; 2019.VIII.9, Shi HL & Liu YZ lgt.”; 17 males and 18 females, “China Jilin province, Antu County, north slope of Changbai mountain, 42.0797N, 128.0637E, 2000m, 2011.VII-VIII, Zou Y lgt.”; 9 males and 3 females, “China Jilin province, Antu County, north slope of Changbai mountain, 42.0877N, 128.0738E, 1820m, 2011.VII-VIII, Zou Y lgt.”; 6 males and 3 females, “China Jilin province, Antu County, north slope of Changbai mountain, 42.0885N, 128.0696E, 1950m, 2011.VII-VIII, Zou Y lgt.”; 11 males, “China Jilin province, Antu County, north slope of Changbai mountain, 42.0888N, 128.0703E, 1750m, 2011.VII-VIII, Zou Y lgt.”; 9 males and 5 females, “China Jilin province, Antu County, north slope of Changbai mountain, 42.0947N, 128.0675E, 1740m, 2011.VII-VIII, Zou Y lgt.”; 12 males and 5 females, “China Jilin province, Antu County, north slope of Changbai mountain, 42.1192N, 128.1047E, 1730m, 2011.VII-VIII, Zou Y lgt.”; 3 males and 1 female, “China Jilin province, Antu County, north slope of Changbai mountain, 42.1797N, 128.1375E, 1520m, 2011.VII-VIII, Zou Y lgt.”; 3 males and 2 females, “China Jilin province, Antu County, north slope of Changbai mountain, 42.0573N, 128.0669E, 1990m, 2012.VII-VIII, Zou Y lgt.”; 15 males and 12 females, “China Jilin province, Antu County, north slope of Changbai mountain, 42.0575N, 128.0656E, 1960m, 2012.VII-VIII, Zou Y lgt.”; 26 males and 10 females, “China Jilin province, Antu County, north slope of Changbai mountain, 42.0797N, 128.0637E, 2000m, 2012.VII-VIII, Zou Y lgt.”; 3 males and 5 females, “China Jilin province, Antu County, north slope of Changbai mountain, 42.0877N, 128.0738E, 1820m, 2012.VII-VIII, Zou Y lgt.”; 7 males and 3 females, “China Jilin province, Antu County, north slope of Changbai mountain, 42.0885N, 128.0696E, 1950m, 2012.VII-VIII, Zou Y lgt.”; 3 males and 6 females, “China Jilin province, Antu County, north slope of Changbai mountain, 42.0888N, 128.0703E, 1750m, 2012.VII-VIII, Zou Y lgt.”; 9 males and 3 females, “China Jilin province, Antu County, north slope of Changbai mountain, 42.0947N, 128.0675E, 1740m, 2012.VII-VIII, Zou Y lgt.”; 11 males and 10 females, “China Jilin province, Antu County, north slope of Changbai mountain, 42.1192N, 128.1047E, 1730m, 2012.VII-VIII, Zou Y lgt.”; 1 male and 3 females, “China Jilin province, Antu County, north slope of Changbai mountain, 42.1209N, 128.1073E, 1620m, 2012.VII-VIII, Zou Y lgt.”; 1 male and 1 female, “China Jilin province, Antu County, north slope of Changbai mountain, 42.1714N, 128.1347E, 1600m, 2012.VII-VIII, Zou Y lgt.”; 1 male and 2 females, “China Jilin province, Antu County, north slope of Changbai mountain, 42.1797N, 128.1375E, 1520m, 2012.VII-VIII, Zou Y lgt.”; 1 female, “ Jilin province, the Changbai mountain Tianchi, 2000m, 1987.VII.22, Yu Peiyu lgt.”; 1 male, “20100818, OH-15-S56”.

#### Diagnosis.

Small sized species in this subgenus; body dark reddish brown; fifth tarsomere without seta on ventral side; median lobe of aedeagus stout, ventral surface without tubercle, apical portion simple, not distinctly deflected to venter or left; endophallus strongly directed ventrally, gonopore opened to the ventral-base of aedeagus; two endophallic lobes present, both spherical; lp a little larger than la.

#### Comparison.

*Pterostichusmicropoides* sp. nov. is most similar to *P.microps* Heyden in its external and aedeagal features: both species have the median lobe of aedeagus without ventral tubercle and not apically dilated. But these two species also differ in their male genitalia: in *P.micropoides* sp. nov., the median lobe of aedeagus is only very slightly deflected ventrally at the apical fourth (Figs 7, 8), while in *P.microps* it is more distinctly deflected ventrally at the apical fourth (Fig. 20). The endophallus of these two species is also very similar: they both curved ventrally with only two endophallic lobes present. But they are different in the orientation of endophallus and shape of lobes: in *P.micropoides* sp. nov., the endophallus is generally directed ventrally, gonopore opened to the ventral-basal direction; the endophallic lobes with la and lp both spherical, lp a little larger than la (Figs 22, 23); while in *P.microps*, the endophallus directed apical-ventrally, gonopore opened to the basal direction; the endophallic lobes with la strongly compressed, much smaller than lp (Fig. 21).

From the external features, *P.micropoides* sp. nov. can be readily distinguished among all Chinese species of *Koreonialoe* for its smaller size (BL 13.7–15.7 mm versus 16.8–21.6 mm in other species) and dorsally reddish brown (a little darker in *P.syleus*, much darker in other two species). Besides, *P.micropoides* sp. nov. is a little different from other three Chinese species in the pronotum punctures: in *P.micropoides* sp. nov., pronotum disc with sparser fine punctures, area anterior to the transversal sulci completely impunctate; but in other species, pronotum disc with much denser fine punctures, area anterior to the transversal sulci distinctly punctate.

#### Description.

BL 13.7–15.7 mm, BW 5.4–6.7 mm. Body form robust, dorsally dark reddish brown, elytra opaque, without metallic luster. Head large, widest at temporae; frons smooth; frontal grooves shallow; temporae strongly swollen, a little longer than eyes; eyes small and hemispherical. Pronotum strongly cordate, disc with relative sparse fine punctures, area anterior to sub-anterior transversal sulci impunctate; PW/HW = 1.31–1.36, PW/PL = 1.42–1.46, widest near anterior third; anterior margin slightly wider than posterior margin, PAW/PBW = 1.14–1.18. Lateral margins slightly arched from anterior angles to the middle, strongly sinuate and then nearly straight before posterior angles; posterior angles rectangular; mid-lateral setae present at anterior fifth of lateral margins; lateral expansions equal width at anterior and posterior portion. Basal foveae with inner and outer grooves faintly defined and partly fused, forming deep depression between them, outer groove slightly shorter than inner one; middle area between two basal foveae longitudinal rugose. Elytra oblong, shoulders widely rounded; basal ridge and lateral margin forming an obtuse angle; elytra 1.53–1.65 times longer than wide. Usually three discal pores present on third interval, all adjoining the second stria: the first one before middle, position of the second one variable, the last one at apical fifth to eighth. Umbilicate series on ninth interval continuous, sparse at middle. Ventral side: metepisternum near smooth; sternite VII of males without secondary sexual modification. Fifth tarsomere without ventral seta. Male genitalia: median lobe of aedeagus gradually curved at basal third; ventral surface without a conspicuous tubercle, apical portion not dilated, gradually deflected ventrally (Figs 7, 8); apical lamella very short, rounded-triangular, nearly straight in dorsal view (Figs 13, 14). Endophallus (Figs 22, 23) long, straightly directed ventrally, gonopore opened to ventral-basal direction of aedeagus; two distinct lobes recognized: left apical lobe (**la**) small, spherical, apex not hooked; left preapical lobe (**lp**) larger than la, similar shape as la. Female genitalia typical in this subgenus.

**Figures 1–6.**
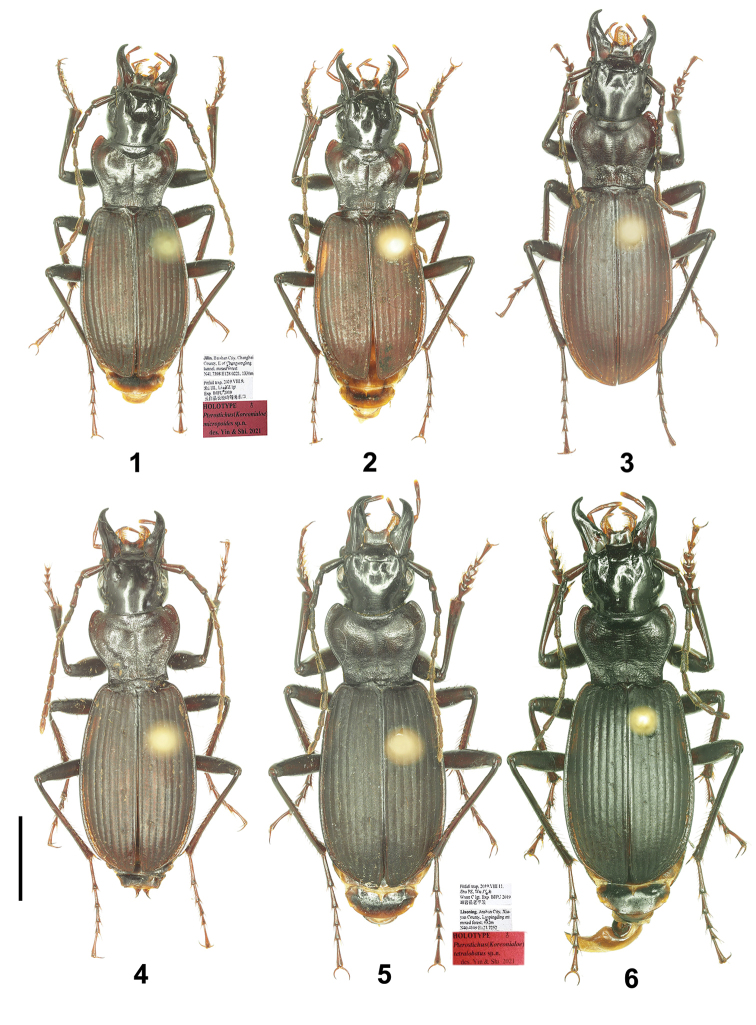
Habitus of Pterostichus (Koreonialoe) spp. from China **1***P.micropoides* sp. nov., Holotype. **2***P.micropoides* sp. nov., male, Paratype from Hunchun city, Jilin **3***P.syleus* Kirschenhofer, a male from Fengcheng city, Liaoning **4***P.syleus* Kirschenhofer, a female from Zhuanghe city, Liaoning 5 *P.quadrilobatus* sp. nov., Holotype **6***P.bellatrix* Tschitschérine, a male from Changbai county, Jilin. Scale bar: 5mm.

**Figures 7–12.**
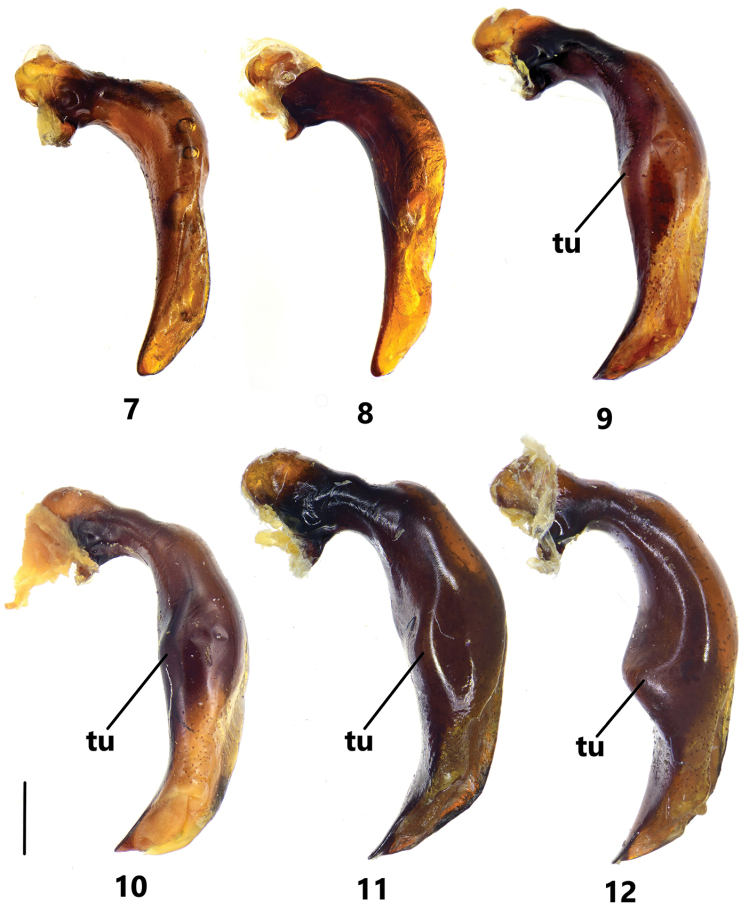
Male genitalia of Pterostichus (Koreonialoe) spp. from China, left lateral view of median lobe of aedeagus **7***P.micropoides* sp. nov., Holotype **8***P.micropoides* sp. nov., Paratype from Changbai county, Jilin **9***P.syleus* Kirschenhofer, a male from Fengcheng city, Liaoning **10***P.syleus* Kirschenhofer, a male from Shedao Island, Liaoning **11***P.quadrilobatus* sp. nov., Holotype **12***P.bellatrix* Tschitschérine, a male from Changbai county, Jilin. Scale bar: 1 mm.

**Figures 13–18.**
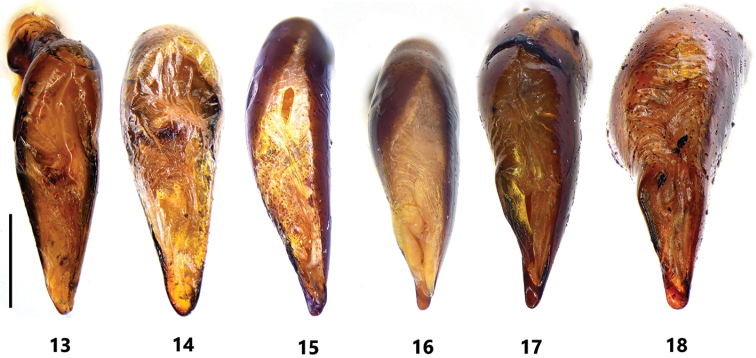
Male genitalia of Pterostichus (Koreonialoe) spp. from China, dorsal view of median lobe of aedeagus **13***P.micropoides* sp. nov., Holotype **14***P.micropoides* sp. nov. Paratype from Changbai county **15***P.syleus* Kirschenhofer, a male from Fengcheng city, Liaoning **16***P.syleus* Kirschenhofer, a male from Shedao Island, Liaoning **17***P.quadrilobatus* sp. nov., Paratype from Laopingding, Liaoning **18***P.bellatrix* Tschitschérine, a male from Changbai county, Jilin. Scale bar: 1 mm.

**Figures 19–21.**
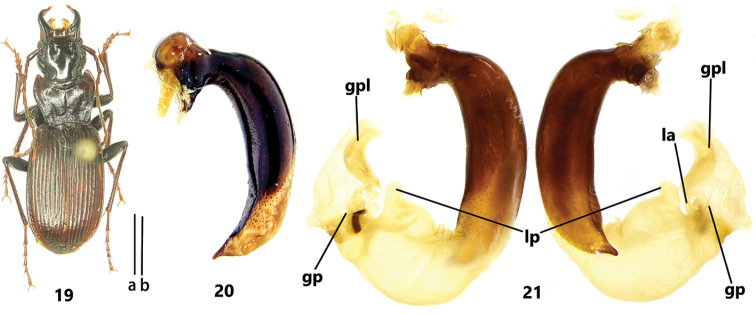
Pterostichus (Koreonialoe) microps Heyden, a male labeled as "Russia, Primorye Terr. Lazovsky reserve, Krodon America, 2002.IV.25, J. Sundukov leg" **19** habitus **20** male genitalia, dorsal view of median lobe, scale bar: 1 mm **21** endophallus, left lateral view and right lateral view. Scale bars: 5 mm (**a** for Fig. 19); 1 mm (**b** Figs 21, 22).

#### Distribution.

This species is relatively widespread along the Changbai mountain range of Jilin province, the border between China and North Korea. (Fig. 36, green)

#### Etymology.

The name of this new species is derived from *P.microps* from the Far East of Russia and a Greek suffix “-oides” meaning alike. It alludes to its similarity to this species.

### 2. Pterostichus (Koreonialoe) tetralobatus sp. nov.

Taxon classificationAnimaliaColeopteraCarabidae

F18AC53C-521F-5055-8AAA-BDCCD6E668F6

http://zoobank.org/09F795C5-1F7F-4215-AE25-608CF1272D8C

Figs 5 , 11 , 17 , 26 , 31 , 35

#### Type locality.

China, Liaoning province: Anshan city, Xiuyan county, Laopingding (40.41N, 123.72E, alt 632m).

#### Type material.

***Holotype*:** male, “Liaoning province, Anshan City, Xiuyan County, Laopingding mountain, mixed forest; 40.4109N, 123.7252E, 632m”; “pitfall trap; 2019.VIII.11, Zhu PZ, Wu JY & Wang C lgt.”; “HOLOTYPE ♂ Pterostichus (Koreonialoe) tetralobatus sp. nov., des. Yin & Shi. 2021” [red label]. ***Paratypes***: 1 male and 2 females, the same data as holotype but labeled as paratype.

#### Diagnosis.

Dorsal side nearly black; fifth tarsomere without ventral seta. Median lobe of aedeagus stout, ventral margin slightly tumid forming an inconspicuous tubercle. Endophallus long and thick, directed basal-ventrally; four lobes present: la divided into two sub-lobes, la1 and la2 both cylindrical, la2 smaller than la1, adnate to la1; ra apically hooked.

#### Comparison.

This new species is peculiar among all the Chinese species of subgenus Koreonialoe for the endophallus has four distinct lobes, while all other species have two or three lobes. The left apical lobe divided into two lobes: la2 much smaller than la1, and adnate to la1. *P.tetralobatus* sp. nov. is very similar to *P.syleus* in the sclerotized part of male genitalia, but quite different in their endophallus.

From the external features, *P.tetralobatus* sp. nov. can be distinguished from *P.micropoides* sp. nov. and *P.syleus* for its larger size (BL 20.2–21.6 mm versus 13.7–19.7 mm in other two species) and dorsally nearly black (versus dark brown in other two species). But, the females of *P.tetralobatus* sp. nov. are completely identical to that of *P.bellatrix*. They can be determined only by the allopatric distributions (Fig. 36).

#### Description.

BL 20.2–21.6 mm, BW 7.2–7.6 mm. Body form robust, dorsal surface nearly black, elytra opaque, without metallic luster. Head large, widest at temporae; frons smooth; frontal grooves shallow; temporae strongly swollen, a little shorter than eyes; eyes small and hemisphere; terminal segment of labial palpus fusiform. Pronotum strongly cordate, disc evenly and densely covered with fine punctures, area anterior to sub-anterior transversal sulci well punctate; PW/HW = 1.27–1.31, PW/PL = 1.58–1.62, widest near anterior third; anterior margin a little wider than posterior margin, PAW/PBW = 1.09–1.14. Lateral margins slightly arched from anterior angles to the middle, strongly sinuate and then nearly straight before posterior angles; posterior angles rectangular; mid-lateral setae present at anterior fifth of lateral margins; lateral expansions equal width anteriorly and posteriorly. Basal foveae with inner and outer grooves faintly defined and partly fused, forming deep depression between them, outer groove slightly shorter than inner one; middle area between two basal foveae longitudinal rugose. Elytra oblong, shoulders widely rounded; basal ridge and lateral margin forming an obtuse angle; elytra 1.58–1.65 times longer than wide. Usually three discal pores present on third interval, all adjoining the second stria; the first one before middle, position of the second one variable, the last one at apical fifth to eighth. Umbilicate series on ninth interval continuous, sparse at middle. Ventral side: metepisternum nearly smooth; sternite VII of males without secondary sexual modification. Fifth tarsomere without ventral seta. Male genitalia: median lobe of aedeagus stout, gradually curved at basal fourth, strongly dilated near middle, apical portion abruptly deflected ventrally; ventral surface shallowly tumid near middle, forming an inconspicuous tubercle (Fig. 11); apical lamella narrow, apex rounded-triangular, slightly oblique to the left in dorsal view (Fig. 17). Endophallus (Fig. 26) long and thick, strongly directed basal-ventrally, gonopore opened to ventral-basal direction of aedeagus; four distinct lobes recognized: left apical lobe (**la**) divided into two sub-lobes, left apical lobe I (**la1**) cylindrical, larger than all other three lobes; left apical lobe II (**la2**) same shape as la1 but smaller, adnate to la1; left preapical lobe (**lp**) oblate and small, located behind la1, well separated from it; right apical lobe (**ra**) small, orbicular, apically hooked. Female genitalia typical in this subgenus.

#### Distribution.

This species was only found in the type locality, Anshan, Liaoning Province, Laopingding mountain. (Fig. 36, red)

#### Etymology.

The scientific name of the new species is composed of two Greek root: “*tetr*-” meaning four and “*lobat*-” meaning lobe. The new species is named for its endophallus with four lobes which is special in this subgenus.

### 3. Pterostichus (Koreonialoe) syleus

Taxon classificationAnimaliaColeopteraCarabidae

Kirschenhofer, 1997

087E167D-2A59-5740-9802-6EED663B2B18

Figs 3 , 4 , 9 , 10 , 15 , 16 , 24 , 25 , 29 , 33

Pterostichus (Koreonialoe) syleus Kirschenhofer, 1997: 694 (Holotype deposited in Naturhistorisches Museum Wien; type locality: “Shi-fang Ding”, sg. Nialoe); Sasakawa et al. 2013: 430 (based on the misidentification of an unknown species).

#### Material examined.

(8 males and 16 females): 4 males, “China, Liaoning, Dandong city, Saima town; Mixed forest, 41.0143N, 124.3020E”; “Day, 2008.VIII.15, pitfall trap, Shan HC lgt.”; 1 male, “Liaoning province, Dalian City, Snake island; 2012, Shi JS lgt.”; 2 males, “China, Liaoning, Benxi City, Guanmenshan mountain; 41.5644N, 123.5779E, 530m, 2011.VIII.20, night, Huang XL lgt.”; 1 male, “China, Liaoning province, Benxi City, Benxi county, Guanmenshan mountain; 41.5644N, 123.5779E, 530m, 2011.VIII.23, day, Huang XL lgt.”; 2 males and 5 females, “China, Liaoning province, Zhuanghe City, Buyunshan mountain; 40.0862N, 122.7234E, pitfall trap, 1133m”; “2019.VIII.15, Zhu PZ & Wang C lgt.”; 6 females, “China, Liaoning, Fushun city, Qingyuan county, Nankouqian town; mixed forest; 2020.VIII, local collector.”; 2 females, “Liaoning province, Baishilazi, 40.9394N, 124.8025E, 567m, 2015.VIII.14, mixed forest”; 1 female, “Liaoning province, Baishilazi, 40.9385N, 124.7891E, 713m, 2015.VIII.15, broadleaved mixed forest”; 2 females, “Liaoning province, Baishilazi, 40.9412N, 124.7986E, 591m, 2015.IX.12, mixed forest”.

#### Diagnosis.

Dorsally dark reddish brown; fifth tarsomere without ventral seta. Median lobe of aedeagus stout, ventral surface slightly tumid forming an inconspicuous tubercle. Endophallus elongate, directed basal-ventrally, gonopore opened to the ventral-basal direction of aedeagus; three lobes present: la and lp both oblate, without hook at apex, similar in size and well separate.

#### Comparison.

From the sclerotized features of male genitalia, *P.syleus* is most similar to *P.tetralobatus* sp. nov. for they both have an inconspicuous tubercle on the ventral margin of median lobe. But they are quite different in the endophallus. *P.syleus* is distinguishable from *P.tetralobatus* sp. nov. for the endophallus with la not divided and ra only very faintly defined, while in the latter species with la divided into two sub-lobes, and ra well defined.

From the external features, *P.syleus* can be distinguished from *P.micropoides* by its slightly larger size (BL 16.8–19.7 mm versus 13.7–15.7 mm), and from *P.bellatrix* and *P.tetralobatus* by the smaller size (BL 16.8–19.7 mm versus 19.7–21.6 mm) and lighter color (dark reddish brown versus nearly black).

#### Description.

BL 16.8–19.7 mm, BW 6.7–7.2 mm. Body form robust, dorsally dark reddish brown, elytra opaque, without metallic luster. Head large, widest at temporae; frons smooth; frontal grooves shallow; temporae strongly swollen, a little shorter than eyes; eyes small and hemisphere; terminal segment of labial palpus fusiform. Pronotum strongly cordate, disc evenly and densely covered with fine punctures, area anterior to sub-anterior transversal sulci well punctate; PW/HW = 1.26–1.32, PW/PL = 1.46–1.51, widest near anterior third; anterior margin a little wider than posterior margin, PAW/PBW = 1.19–1.21. Lateral margins slightly arched from anterior angles to the middle, strongly sinuate and then nearly straight before posterior angles; posterior angles rectangular; mid-lateral setae present at anterior fifth of lateral margins; lateral expansions equal width anteriorly and posteriorly. Basal foveae with inner and outer grooves faintly defined and partly fused, forming deep depression between them, outer groove slightly shorter than inner one; middle area between two basal foveae longitudinal rugose. Elytra oblong, shoulders widely rounded; basal ridge and lateral margin forming an obtuse angle; elytra 1.59–1.64 times longer than wide. Usually three discal pores present on third interval, all adjoining the second stria; the first one before middle, position of the second one variable, the last one at apical sixth to eighth. Umbilicate series on ninth interval continuous, sparse at middle. Ventral side: metepisternum nearly smooth; sternite VII of males without secondary sexual modification. Fifth tarsomere without ventral seta. Male genitalia: median lobe of aedeagus stout, gradually curved at basal third, slightly dilated near middle, apical portion abruptly deflected ventrally; ventral surface shallowly tumid near middle, forming an inconspicuous tubercle (Figs 9, 10); apical lamella narrow, apex rounded-triangular, very faintly oblique to the left in dorsal view (Figs 15, 16). Endophallus (Figs 24, 25) long, directed basal-ventrally, gonopore opened to ventral-basal direction of aedeagus; three endophallic lobes recognized: right apical lobe (**ra**) faintly defined, close to gonopore; left apical lobe (**la**) oblate, apex not hooked; left preapical lobe (**lp**) similar shape and size as la, these two lobes well separated. Female genitalia typical in this subgenus.

#### Distribution.

Widely distributed in the eastern part of Liaoning province, along the Qianshan mountain range. (Fig. 36, blue)

#### Remarks.

Sasakawa et al. (2013) redescribed this species and illustrated its endophallus based on a specimen from the type locality (Shi-Fang-Ding, the highest peak of Baishilazi nature reserve). However, their description and illustrations do not accord with the original literature (Kirschenhofer 1997) or our examined specimens from the type locality (Baishilazi). Sasakawa et al. (2013) indicated that *P.syleus* is the only species of *Koreonialoe* with the fifth tarsomeres setose beneath, but in the original description Kirschenhofer (1997) mentioned this species has: “*Klauenglieder unterseits glatt*”. Moreover, compared with the line drawing of the male genitalia of *P.syleus* (Kirschenhofer 1997: Fig. 23), specimen described by Sasakawa et al. (Sasakawa et al. 2013: Fig. 2D) is different in the sclerotized part of male genitalia in the ventral tubercle a little larger and the apical portion of median lobe less deflected ventrally in lateral view. Thus, although the specimen described by Sasakawa et al. (2013) is exactly from the type locality of *P.syleus*, we thought it should belong to an unknown new species different from *P.syleus* and all other known species of *Koreonialoe*. Besides the differences mentioned above, these two species are also different in their endophallus: *P.syleus* has the endophallus more strongly directed ventro-basally, and lp is oblate, with apex not hooked (Figs 24, 25); but the species by Sasakawa et al. (2013) has endophallus less directed ventral-basally, and lp is coniform, with distinctly hooked apex (Sasakawa et al. 2013: Fig. 2D).

### 4. Pterostichus (Koreonialoe) bellatrix

Taxon classificationAnimaliaColeopteraCarabidae

(Tschitschérine, 1895)

66079427-D516-5C98-984E-20EF8023F913

Figs 6 , 12 , 18 , 27 , 28 , 32

Pterostichus (Koreonialoe) bellatrix Tschitschérine, 1895: 169 (Syntypes deposited in Zoological Institute, Russian Academy of sciences, St Petersburg, Russia; type locality: North Korea, sg. Feronia); Jedlička 1962: 271 (misspelled as P.bellator, sg. Lianoe); Kwon and Lee 1986: 28 (misspelled as P.bellator, sg. Nialoe); Park and Kwon 1996: 96 (misspelled as P.bellator); Sasakawa 2005a: 1209 (misspelled as P.bellator); Sasakawa et al. 2013: 430; Park and Park 2013: 66. **New record from China**
Pterostichus klickai Jedlička, 1931: 104. (Holotype deposited in Narodni Muzeum Prirodovedecke Muzeum, Prague, Czech Republic, Type locality: Seishin, Korea); Cho 1957: 194; Jedlička 1962: 272 (sg. Lianoe); Kwon and Lee 1986: 28; Nemoto 1988: 40; Bousquet 2003: 497 (synonymized with P.bellatrix).

#### Material examined.

(6 males and 1 female): 1 male, “China Jilin province, Baishan City, Changbai County, E of Changsongling tunnel; Mixed forest, 41.7398N, 128.0221, 1330m”; “pitfall trap; 2019.VIII.9, Shi HL & Liu YZ lgt.”; 4 males and 1 female, “China Jilin province, Baishan City, Fusong county, W of Changsongling tunnel; mixed forest, 41.7798N, 127.9400E, 1577m”; “pitfall trap, 2019.VIII.9, Shi HL & Liu YZ lgt.”; 1 male, “Jilin province, Changbai Mountain Hot Spring, 1982.VII.13, Liao Suyi lgt.”.

#### Diagnosis.

Dorsal surface nearly black; fifth tarsomere without ventral seta. Median lobe of aedeagus stout, ventral margin strongly tumid forming a conspicuous cuneate tubercle anterior to middle. Endophallus directed basal-ventrally, gonopore opened to the ventral-basal direction of aedeagus; three lobes present: la and lp similar in size, la a little hooked apically, ra faintly defined.

#### Comparison.

*P.bellatrix* is similar to *P.syleus* and *P.tetralobatus* in their external features, but can be distinguished from the latter two species by the more conspicuous ventral tubercle on male genitalia. Another species, *P.woongbii* from South Korea, also has a conspicuous ventral tubercle on male genitalia, but is quite different from *P.bellatrix* in the ventral tubercle located near base of median lobe, and endophallus short and straight.

From the external features, *P.bellatrix* can be distinguished from *P.micropoides* sp. nov. and *P.syleus* for its larger size (BL 19.7–21.2 mm versus 13.7–19.7 mm in other two species) and dorsally nearly black (versus dark reddish brown in other two species). But, the females of *P.bellatrix* are completely identical to that of *P.tetralobatus* sp. nov. They can be determined only by the allopatric distributions (Fig. 32).

#### Description.

BL 19.7–21.2 mm, BW 6.3–6.6 mm. Body form robust, dorsally dark brown, nearly black, elytra opaque, without metallic luster. Head large, widest at temporae; frons smooth; frontal grooves shallow; temporae strongly swollen, a little shorter than eyes; eyes small and hemispherical; terminal segment of labial palpus fusiform. Pronotum strongly cordate, disc evenly and densely covered with fine punctures, area anterior to sub-anterior transversal sulci well punctate; PW/HW = 1.23–1.27, PW/PL = 1.53–1.56, widest near anterior third; anterior margin a little wider than posterior margin, PAW/PBW = 1.16–1.18. Lateral margins slightly arched from anterior angles to the middle, strongly sinuate and then nearly straight before posterior angles; posterior angles rectangular; mid-lateral setae present at anterior fifth of lateral margins; lateral expansions equal width anteriorly and posteriorly. Basal foveae with inner and outer grooves faintly defined and partly fused, forming deep depression between them, outer groove slightly shorter than inner one; middle area between two basal foveae longitudinal rugose. Elytra oblong, shoulders widely rounded; basal ridge and lateral margin forming an obtuse angle; elytra 1.53–1.56 times longer than wide. Usually three discal pores present on third interval, all adjoining the second stria; the first one before middle, position of the second one variable, the last one at apical sixth to eighth. Umbilicate series on ninth interval continuous, sparse at middle. Ventral side: metepisternum nearly smooth; sternite VII of males without secondary sexual modification. Fifth tarsomere without ventral seta. Male genitalia: median lobe of aedeagus stout, gradually curved at basal third, apical portion gradually deflected ventrally; ventral surface strongly tumid near middle, forming a conspicuous cuneate tubercle a little anterior to the middle (Fig. 12); apical lamella short and wide, apex rounded, not oblique to the left in dorsal view (Fig. 18). Endophallus (Fig. 27) long, directed basal-ventrally, gonopore opened to the ventral-basal direction of aedeagus; three endophallic lobes recognized, all placed near gonopore: left apical lobe (**la**) oblate, apex slightly hooked, forming an oblique upper surface; left preapical lobe (**lp**) similar size as la, apex rounded, well separate from la; right apical lobe (**ra**) faintly defined, nearly spherical. Female genitalia typical in this subgenus.

**Figures 22–27.**
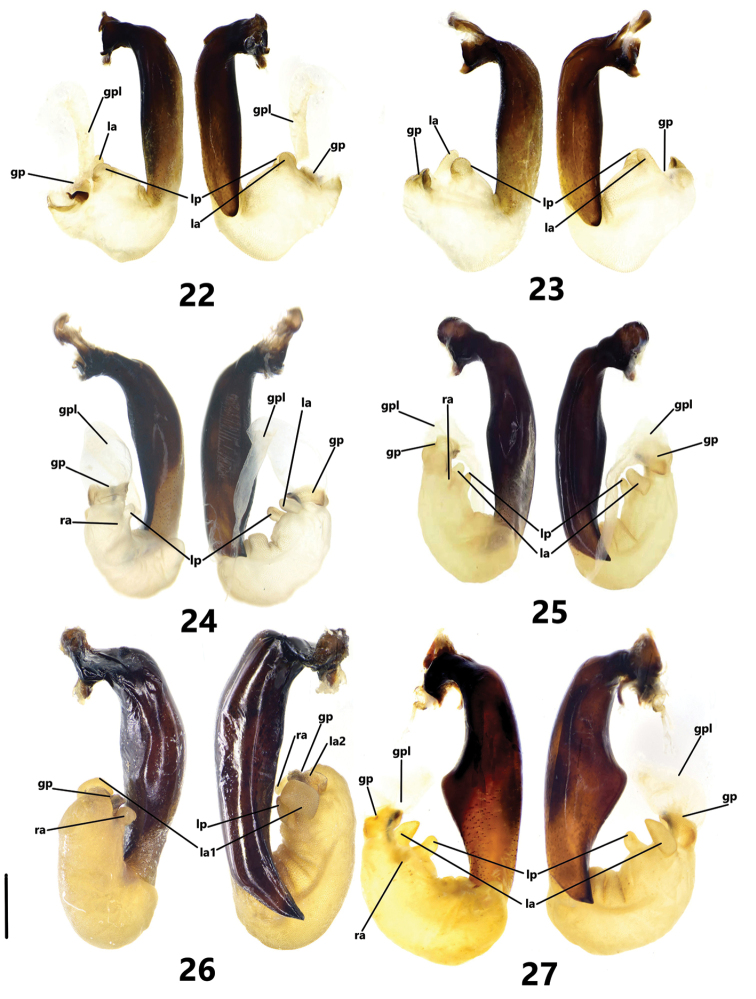
Endophallus of Pterostichus (Koreonialoe) spp. from China, left lateral view and right lateral view **22***P.micropoides* sp. nov., Paratype from Antu city, Jilin **23***P.micropoides* sp. nov., Paratype from Changbaishan mt., Jilin **24***P.syleus* Kirschenhofer, a male from Fengcheng city, Liaoning **25***P.syleus* Kirschenhofer, a male from Shedao Island, Liaoning **26***P.quadrilobatus* sp. nov., Holotype **27***P.bellatrix* Tschitschérine, a male from Changbai county, Jilin. Scale bar: 1 mm.

**Figures 28–35.**
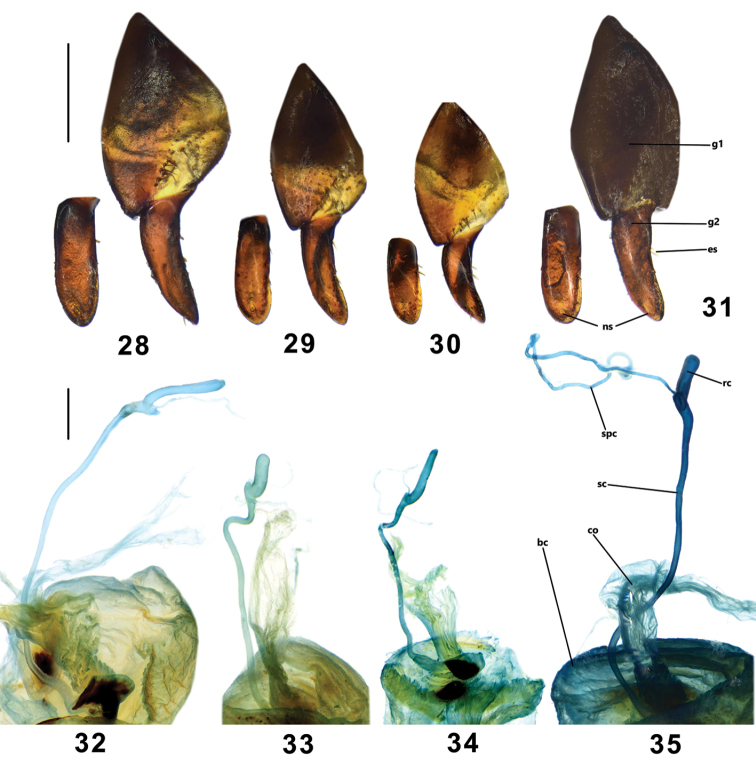
Female genitalia of Pterostichus (Koreonialoe) spp. from China, Figs **28–31** ventral view and inner lateral view of ovipositor. Figs **32–35** female reproductive system. **28, 32***P.tetralobatus*, Paratype from Laopingding, Liaoning **29, 33***P.syleus*, a female from Baishilazi, Liaoning **30, 34***P.micropoides*, female, Paratype from Antu county, Jilin **31, 35***P.bellatrix*, a female from Fusong county, Jilin. Scale bar: 0.5 mm. Abbreviations: g1: gonocoxite I; g2:gonocoxite II; es: ensiform setae; ns, nematiform setae; bc: bursa copulatrix; co: common oviduct; sc: seminal canal; rc: receptaculum; spc: spermathecal canal.

#### Distribution.

**Figure 36.**
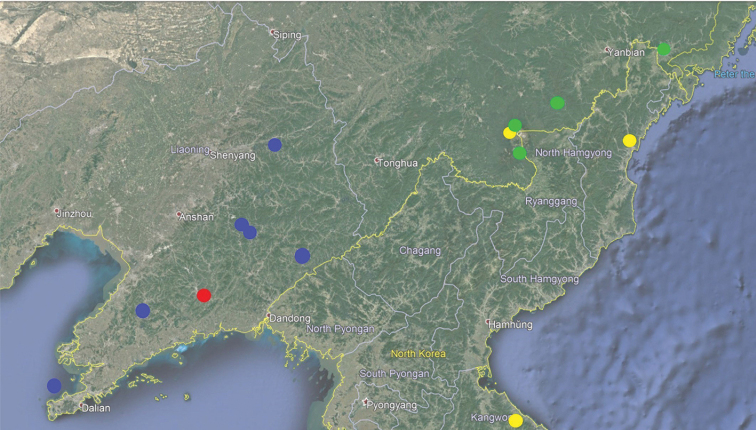
Confirmed distribution of Pterostichus (Koreonialoe) spp. in China: Green: *P.micropoides* sp. nov.; Red: *P.tetralobatus* sp. nov.; Yellow: *P.bellatrix* Tschitschérine; Blue: *P.syleus* Kirschenhofer.

This species is widespread from the southeastern part of Jilin province to South Korea, along the eastern mountains in the Korean Peninsula. (Fig. 36, yellow, distributions in South Korea not shown)

## Supplementary Material

XML Treatment for Koreonialoe

XML Treatment for Pterostichus (Koreonialoe) micropoides

XML Treatment for Pterostichus (Koreonialoe) tetralobatus

XML Treatment for Pterostichus (Koreonialoe) syleus

XML Treatment for Pterostichus (Koreonialoe) bellatrix
